# Knowledge and use of medicinal plants by local specialists in an region of Atlantic Forest in the state of Pernambuco (Northeastern Brazil)

**DOI:** 10.1186/1746-4269-1-9

**Published:** 2005-11-01

**Authors:** Luiz Rodrigo Saldanha Gazzaneo, Reinaldo Farias Paiva de Lucena, Ulysses Paulino de Albuquerque

**Affiliations:** 1 Departamento de Biologia, Área de Botânica, Laboratório de Etnobotânica Aplicada, Universidade Federal Rural de Pernambuco, Rua Dom Manoel de Medeiros s/n, Dois Irmãos, Recife, Pernambuco, 52171-030, Brazil

**Keywords:** ethnobotany, Atlantic Forest, medicinal plants, traditional knowledge

## Abstract

The study of local knowledge about natural resources is becoming increasingly important in defining strategies and actions for conservation or recuperation of residual forests. This study therefore sought to: collect information from local populations concerning the use of Atlantic Forest medicinal plants; verify the sources of medicinal plants used; determine the relative importance of the species surveyed, and; calculate the informant consensus factor in relation to medicinal plant use. Data was obtained using semi-structured forms to record the interviewee's personal information and topics related to the medicinal use of specific plants. The material collected represent 125 plants, distributed among 61 botanical families, with little participation of native plants. This study demonstrated that local people tend to agree with each other in terms of the plants used to treat blood-related problems, but cite a much more diverse group of plants to treat problems related to the respiratory and digestive systems – two important categories in studies undertaken in different parts of the world. The local medicinal flora is largely based on plants that are either cultivated or obtained from anthropogenic zones, possibly due to the use and access restrictions of the legally protected neighboring forest. Despite these restrictions, the species with the highest use-value by this community was *Pithecellobium cochliocarpum *(Gomez) Macb., a native plant of the Atlantic Forest.

## Introduction

Fifteen percent of Brazil was once covered by Atlantic Forest, and although less than 5% of the original forest remains today [1], it is still one of the highest biodiversity areas on the planet [2]. This forest also demonstrates extremely high rates of endemism: up to 74.4% for bromeliads, 55% for trees, and 64% for palms [1,3], and we can infer that there is yet much to be studied and discovered within this ecosystem, especially in terms of its useful resources.

The Atlantic Forest ecosystem has been the victim of successive economic cycles since colonial times (brazilwood, sugarcane, mining, coffee, and cattle), especially in areas very near the coast. Today, large cities (50% of the Brazilian population) are located in areas once covered by these forests, and 80% of the GNP is produced there [1]. For these reasons, innumerous species have disappeared since Brazil's discovery, and continue to disappear without having been recorded; and with them, important information related to ecology, pharmacology, botany, and other fields, may vanish completely.

Rural communities are considered to be the most neglected area in terms of ethnobotanical studies [4]. Despite the availability of modern medicines, most agro-cultural communities (either by choice or for lack of economic resources) still use and detain an extensive pharmacopoeia of native plants [4].

The study of local knowledge about natural resources is becoming increasingly important in defining strategies and actions for conservation or recuperation of residual forests. This study therefore sought to: collect information from local populations concerning the use of Atlantic Forest medicinal plants; verify the sources of medicinal plants used; determine the relative importance of the species surveyed, and; calculate the informant consensus factor in relation to medicinal plant use.

## Materials and methods

### The study area and its inhabitants

**Figure 1:**
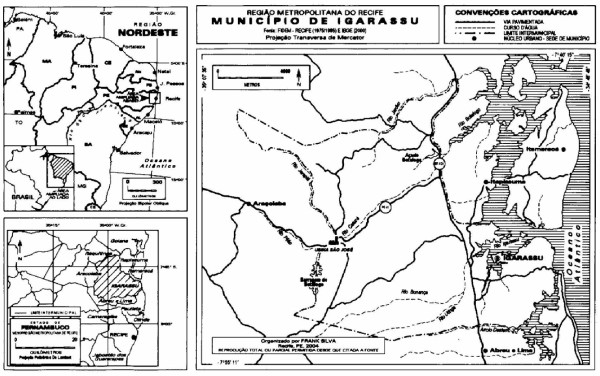
Place of ethnobotanical data collection of the medicinal plants cited by the population in the municipality of Igarassu (Northeast Brazil).

This field study was undertaken with the aid of the "Três Ladeiras" community in the municipality of Igarassu, Pernambuco State, in northeastern Brazil. Igarassu is located in the mesoregion of the Recife metropolitan area (the state capital) and in the Itamaracá microregion (Figure 1). The municipal seat is located 26 km from the state capital [5] at 20 m above sea level (7° 50' 20"S and 35° 00' 10"W). The municipality has a total area of 304.2 km^2^, a population of 72,990 (219.9 inhabitants/km^2^), and it is 74.9% urbanized. The regional climate is humid, with a mean annual temperature of 27°C. Average annual rainfall is 2,000 mm, with a moderate water deficit in the summer [6].

### The Rural Inhabitants

The community studied is part of the "Usina São José" sugarcane refinery, which employs most of the male population. The community is surrounded by Atlantic Forest fragments that belong to the refinery's ecologic reserve [7] (Fig. 1). The fragments occupy a total area of 210 hectares, with altitudes varying from 50 to 140 m. The number of people employed by the refinery oscillates during the year, increasing and decreasing according to the seasons and the periods of land preparation, planting, and harvesting. It is common to find families cultivating small plantations that serve as a nutritional and/or economic reserve between periods of employment activity. There is a 323.3 ha forest southeast of the refinery, which represents 0.80% of the total area of the municipality. The forest is part of the Botafogo River Basin source protection area, according to law N° 9860, dated August 12, 1986. The purpose of this reserve is to protect the landscape, soil, and the water basin [8].

The community views itself as a coherent group, and is formed basically by active or retired workers of the local sugar cane refineries. The women of the community carry out almost all of the activities related to caring for the family and their property. Their are two public schools and a small Peruvian cherry ("*acerola*") plantation that produces fruit pulp and provides job opportunities for youths during the harvest season. There is no basic sanitation, and a single health care center treats only simple health problems; patients requiring more intensive care are removed to hospitals in Igarassu.

### Data Collection

Information on the use of medicinal plants was collected for one year (2003) with the help of rural dwellers of the "Três Ladeiras" community. During this period, door-to-door visits were made in order to attempt to identify local people with a specialized knowledge of medicinal plant use [9]. As such, the sampling was intentionally non-random [10], under the assumption that local specialists would provide more specific and higher quality information concerning medicinal plants. Data was obtained using semi-structured forms to record the interviewee's personal information and topics related to the medicinal use of specific plants. During the first contacts with the local population, a specialist was identified by the inhabitants themselves. A specialist is defined as "a person recognized by the community as having deep knowledge about the uses of native and/or introduced plants in manufacturing remedies and in promoting cures". Using the snowball method [11], names of other specialists were then obtained. Six local specialists were eventually interviewed (three men and three women), aged 51–102. All the people identified as specialists had been living in the community for at least 30 years and were frequently sough out by other community members for advice on the use of plants. The men had all been woodsmen or hunters, although these activities are currently prohibited. The women were housewives who had acquired a broad knowledge of medicinal plants by way of experimentation and trading information with relatives or people from the surrounding communities

### Data Analysis

The first step employed in the data analysis calculating the informant consensus factor (ICF) [12]. ICF values will be low (near 0) if plants are chosen randomly, or if informants do not exchange information about their use. Values will be high (near 1) if there is a well-defined selection criterion in the community and/or if information is exchanged between informants.

The ICF is calculated as follows: number of use citations in each category (n_ur_) minus the number of species used (n_t_), divided by the number of use citations in each category minus one:



All citations were placed into one of 14 categories: undefined pains or illnesses; skin and subcutaneous tissues; diseases of the endocrine glands, metabolism, and nutrition; blood and hematopoietic organs; skeletal, muscle, and connective tissues; infectious and parasite-related diseases; neoplasies; problems of the circulatory system; problems of the digestive system; problems of the genitourinary system; problems of the nervous system; problems of the respiratory system; problems of the sensorial system – ear; and problems of the sensorial system – eye.

The use value (adapted from the proposal of Phillips et al. [13]), a quantitative method that demonstrates the relative importance of species known locally, was also calculated:

UV = ΣU/n

where: UV = use value of a species; U = number of citations per species; n = number of informants

All of the material collected was processed, identified with the aid of specialists, and subsequently deposited in the PEUFR herbarium of the Biology Department of the Federal Rural University of Pernambuco. All material was collected with the help of local informants.

## Results

### Local specialists and medicinal plants

The plants collected represent 125 plants, distributed among 61 botanical families (e.g. see additional file 1). Lamiaceae (12 spp.) was best represented in terms of the number of species, followed by Asteraceae (6 spp.), Myrtaceae (5 spp.), Arecaceae (5 spp.), and Poaceae (5 spp.). When analyzing the number of citations for the plant parts used to prepare local remedies, a preference for the use of leaves becomes noticeable. The use of teas had the highest relative value (42%), followed by the use of syrups (20%), and *in natura *(16%).

Plants cited are listed by collection locality (anthropogenic zones or forest) and degree of management (cultivated or non-cultivated). It was observed that 54.5% of the species used are cultivated, while native and/or weed plants composed the non-cultivated category. In terms of where the plants were collected, 82.7% were from areas backyards and small farms, while only 17.3% were gathered from inside the forest. Most of the non-cultivated plants were weed, with little participation of native plants.

The species with the highest use-value was *Pithecellobium cochliocarpum *(Gomez) Macbr, popularly known as "barbatenon", with an UV of 1.8. Its main importance resides in its attributed healing effects on wounds. It is used externally, in the form of an alcoholic bark extract. *Alpinia zerumbet *(Pers.) Burt. ex R. M. Smith ("colônia") had the second highest UV (1.6). This species is frequently used as an ornamental plant in home gardens. Its main medicinal uses are to treat coughs (flower, leaf, and/or a root syrup), and headaches (a lightly heated leaf is applied to the forehead).

Two species shared the third highest use value (1.3): *Hymenaea martiana *Hayne ("jatobá") and *Aeolanthus suaveolens *Mart. ex Spreng. ("macassá"). *H. martiana *was cited for the treatment of blows to the body, inflammations, and rheumatism. It is principally prepared as an alcoholic extract of the fruit, using the local distilled drink ("cachaça") or wine. *A. suaveolens *is well known among the interviewees as being capable of treating general pains, especially earaches (for which it is applied *in natura*, as an earplug).

Cited for the treatment of inflammations, *Schinus terebinthifolius *Raddi ("aroeira"), *Boerhavia diffusa *L ("pega-pinto"), and *Borreria verticillata *G. F. W. Meyer ("vassoura de botão") all had a use value of 1.1. *Protium heptaphyllum *(Aubl.) March. ("amescla") is used to treat tooth and headaches, and *Solanum panicultum *L. ("jurubeba branca") to treat several ailments (such as indigestion, anemia, hepatitis, kidney, and spleen problems); both had use-values of 1.

### Consensus factor among specialists

The highest ICF values were linked to problems related to the blood and to hematopoietic organs (1.0), and for problems of the sensorial system – ear (0.60). The use category with the lowest ICF value was "diseases of the endocrine glands, metabolism, and nutrition" (0.14). The ICF for infectious and parasite-related diseases was strikingly low (0.27) – the result of the use of a number of species that was almost as varied as the number of citations. A more detailed description of each category follows (see Table 1).

**Table 1:** Informant Consensus Factor by corporal systems categories or diseases.

**Category**	**Species**	**(%) All Species**	**Use citations**	**(%) All use citations**	**ICF**
Undefined pains or illnesses	42	32.3	68	19.88	0.38
Skin and subcutaneous tissues	16	12.3	34	9.9	0.54
Diseases of the endocrine glands, metabolism and nutrition	7	5.3	8	2.3	0.14
Blood and hematopoietic organs	1	0.7	5	1.4	1.00
Skeletal, muscle and connective tissues	13	10.0	23	6.7	0.45
Infectious and parasite-related diseases	14	10.7	19	5.5	0.27
Neoplasies	1	0.7	1	0.2	0.00
Problems of the circulatory system	7	5.3	10	2.9	0.33
Problems of the digestive system	36	27.6	60	17.5	0.40
Problems of the genitourinary system	24	18.4	34	9.9	0.30
Problems of the nervous system	3	2.3	4	1.16	0.33
Problems of the respiratory system	38	29.2	65	19.0	0.42
Problems of the sensorial system (ear)	3	2.3	6	1.7	0.60
Problems of the sensorial system (eye)	3	2.3	5	1.4	0.50

#### Blood and hematopoietic organs

This category had the highest ICF value (1.0). There were five citations for a single species, demonstrating total agreement on selection criteria among the informants. The main problem treated within this category was hemorrhaging, often caused by accidents involving the perforating or cutting instruments used in sugarcane culture and in subsistence farming. Blood loss is diminished by applying a cloth soaked in a tincture made from the bark of *Pithecellobium cochliocarpum *(Gomez) Macbr ("barbatenon").

#### Problems of the sensorial system – ear

This group obtained the second highest ICF value (0.60). Of all of the plants cited by informants (127), 2.3% (three species) are used to treat medical problems within this group. Only one of the interviewees did not cite an ear-related disease. Earaches were cited by all of the specialists, and there was general agreement in the citations of *Aeolanthus suaveolens *Mart. ex Spreng. as the main remedy. There was also agreement on the use of *Plectranthus sp*. ("hortelã grande") and *Ruta graveolens *L ("arruda"). In all cases, the manner of usage was an earplug made from the leaves (*in natura*).

#### Skin and subcutaneous tissues

With a total of 34 citations (10% of the total), this category had an ICF of 0.54. Its main representative – as in the first category cited – was *Pithecellobium cochliocarpum *(Gomez) Macbr ("barbatenon"), a plant of great importance to the local population.

#### Problems of the sensorial system – eye

This is a heterogeneous group, with an ICF of 0.50. Of the five use-citations, three species are recommended for "tired eyesight" and conjunctivitis. The most cited phytotherapeutic resource was *Ocimum basilicum *L. ("manjericão"), with three citations. Recipes mention the use of an aqueous extract of this plant (obtained as a tea) as an eyewash.

#### Skeletal, muscle, and connective tissues

With an ICF of 0.45, the 23 citations of this group included 10% (13 species) of the total number of plants cited in this study. The most important representatives of this group were *Hymenaea martiana *Hayne ("jatobá"), with four citations, and *Caesalpinia ferrea *Mart. ex Tul. ("jucá"), with three citations. It is interesting to note that in both species the fruit is used, and it is prepared in a very similar fashion – wine is employed to obtain an alcoholic solution for internal use.

#### Problems of the respiratory system

This category had the second highest number of citations (65), only slightly less than the group of undefined pains and illnesses (68). The ICF of this category was 0.42. It is important to note that approximately 32% (42) of the total number of species cited were included within this group, demonstrating a great diversity in the knowledge of medicinal plants for the treatment of respiratory problems. The remedies used are mainly administered in the form of syrups to treat coughing, breathlessness, asthma, bronchitis, and the common cold – maladies that especially affect children, either due to the fragility of their immunological system, or to problems related to their immature respiratory system.

According to the data collected, sugar is often added to the syrup preparation – for in addition to its preservative properties (it increases the osmolality of the solution), its sweet taste masks the bitter and unpalatable taste of some of the herbs.

The documented use of a wide variety of species is in great part due to the practice of using innumerous herbs (sometimes over 15) in recipes for a single syrup, and by the belief that the greater the number of herbs (always in odd numbers) the better the syrup. Another reason for the large number of citations is the frequency of cases of breathlessness and asthmatic problems when the sugarcane fields surrounding the community are burned during the pre-harvest preparations.

#### Problems of the digestive system

This category includes all problems related to organs directly or indirectly linked to digestion, including the teeth and gums. The ICF value for this group was 0.40, considered low. However, if we consider only the citations for toothaches, the ICF value would be nearly 1.0, since there was great agreement on the use of *Aeolanthus suaveolens *Mart. ex Spreng. ("macassá") to treat this complaint.

#### Undefined pains or illnesses

This category includes all citations for undefined pains, and for diseases with unspecific symptoms. The ICF value was 0.38. This group had the highest number of use-citations (68 of a total of 342) as well as species used (42). This result is compatible with the category's diversity of problems.

Once again, *Aeolanthus suaveolens *Mart. ex Spreng. ("macassá") stands out, followed by "verga morta" (an undetermined species) and *Protium heptaphillum *(Aubl.) March. ("amescla"). All of these plants have analgesic uses. *Pfaffia glomerata *(Spreng.) Pederson ("acônito") had the highest number of citations among plants used to treat fevers. *Ruta graveolens *L. ("arruda") stood out for its importance as an anti-inflammatory agent.

#### Problems of the circulatory system

Heart diseases and blood pressure are examples of problems within this category. A total of seven species were cited, with 10 uses. The ICF for this category was low (0.33). Strokes received great attention from the interviewees, totaling 60% of the citations. *Argemone mexicana *L. ("cardo santo") was the species most cited.

#### Problems of the nervous system

An ICF of 0.33 was observed for this category. Fainting and insomnia were the most frequent citations. Half of the citations referred to the calming effects attributed to a tea made from orange tree flowers or leaves (*Citrus sinensis *(L.) Burmann), which is also frequently used to treat insomnia.

#### Problems of the genitourinary system

With 34 citations, this category comprises 24 plant species, with an ICF of 0.30. Women in the community commonly use sitting baths made with tea from the bark of *Boerhavia diffusa *L ("pega-pinto") or *Pithecellobium cochliocarpum *(Gomez) Macbr ("barbatenon").

#### Infectious and parasite-related diseases

The ICF for this category was 0.27. The common cold was frequently cited, followed by worms and hepatitis. A total of 19 disease citations were recorded, and 14 plants species were indicated as cures for these diseases, or as treatments for their symptoms.

#### Diseases of the endocrine glands, metabolism, and nutrition

The most cited diseases were diabetes (metabolism) and anemia (nutrition). Seven plants were cited for eight diseases, and the ICF was correspondingly very low (0.14).

#### Neoplasies

As only a single citation was obtained (for cancer) and therefore this category's ICF appears as 0, as this index requires citations from at least two informants.

## Discussion

There seems to be a tendency for a few plant families to stand out in any pharmacopoeia. In a study by Almeida & Albuquerque [14], the family Lamiaceae was classified as the richest in species citations. The families Lamiaceae and Asteraceae stood out in the studies of Bennett & Prance [15], for together they represented 21% of the 216 species surveyed. Hanazaki *et al*. [2] likewise found that these two families had the greatest number of species cited by fishing communities and in other coastal areas of Atlantic Forest in São Paulo State [16]. These plants are normally herbaceous species that can either be cultivated or occur as weeds. Preference for their use may be related as much to their ready availability, for they are common in disturbed areas [17], as to factors related to their biological activity. Based on evidence and availability theory, Stepp & Moerman [18] suggest that these plants concentrate very active biological compounds as a function of their habit or of their life strategies. Much evidence has accumulated indicating that chemical and ecological factors orient the selection and use of medicinal plants in local communities in all parts of the world [cf. [19,20]].

Another interesting factor is that the cited species generally are indicated to the same illnesses in other localities [2,3,14,17,23,25,28,29]. Moreover, observing the lists of species of other studies, it can be evidenced that the most important species vary of region for region [14,20,23,27], although to verify a set of species, in special the cultivated ones, common in several works, as: *Plectranthus *spp., *Mentha *spp. and *Ocimum *spp.

Another relevant observation relates to the tendency of communities living near humid forests to use plant leaves. In a study undertaken in Barra do Piraí (state of Rio de Janeiro) with merchants and traditional medicine users, Parente & Rosa [21] observed that leaves were the most used category of plant parts. In this study, leaves were used in 42% of the time – over twice the number of citations for the second most used part (the root, at 18%). Similar observations had already been recorded for other communities near forested areas, where vegetation is always green and leaves are abundant [16,22]. On the other hand, communities in dry regions tend to focus their attention on plant parts that are continuously available, such as the bark [23,14]. As the plants in these localities are regularly exposed to long periods of drought and lose their leaves, bark and roots are more often used. This observed difference in usage of plant parts in different areas should be more closely investigated. Zschocke *et al*. [24] suggested, for example, that the use of bark is much more common in tropical regions than in temperate zones. However, as noted above, even in tropical regions the use of bark seems to be most strongly associated with arid and semi-arid zones, reflecting the resources most readily available to these communities. Additionally, the preference towards leaves places more emphasis on the preparation of medicinal teas. Parente & Rosa [21] also found high percentages for teas (51%), baths (39%), and other forms (liquid extracts, infusions, and *in natura *– 10%) due to the great preference for leaves in preparing remedies.

The emphasis on cultivated plants observed in this study was not unexpected. However, it might also have been expected that a larger proportion of the plants cited would be native species in view of the fact that the informants are all specialists having an excellent knowledge of the native resources. Nonetheless, the proportion of non-cultivated to cultivated species used varies among different communities that have access to the resources in the Atlantic Forest [2,25,16]. Amorozo [22] observed a similar situation in the community of Barcarena (state of Pará), where 57.2% of the plants used were cultivated, while only 42.8% were not. Yet, in the same study, Amorozo [22] demonstrated that in the Santo Antonio do Leverger community (state of Mato Grosso) the percentages invert themselves, with non-cultivated plants totaling 56.2% and cultivated plants 43.8%. This result may be due to the fact that Santo Antonio de Leverger is undergoing a process of modernization, resulting in additional anthropogenic alterations in the natural areas where many medicinal plants grow, and causing the devaluation of local culture as well as the loss of traditional practices such as plant cultivation.

In regards to the population studied here, their preference for using plants from anthropogenic zones may be explained by the establishment of the "Usina São José" forest reserve, which does not permit plant collection. This would force the local specialists to rely more on weed and cultivated plants. Local specialists informed us that, in function of the prohibition, they prevent to collect plants in the fragment forest. In this sense, they "opted" to using non-native plants. The ample use of plants derived from disturbed areas is quite common in many parts of the world [26], including areas of Atlantic Forest [17]. This may be explained by easy access to these species, especially when there is little cultural resistance to using such plants. Albuquerque *et al*. [27] counters this idea when he cites the preference of a rural northeastern Brazilian community for native species even when they have easy and rapid access to substitutes in disturbed areas.

In general, the results presented here give rise to two questions. First, what are the implications of the local use of a large number of non-native species? A mixture of native and introduced species in the pharmacopoeias of tropical zone communities is quite common [15], and in view of the complexity of the local knowledge, cannot be simply explained. On one hand, Begossi *et al*. [25] view the question from a conservationist's point of view, as the use of cultivated species reduces pressure on native forest products. On the other hand, in the case of the present study, the use of exotic and cultivated plants is a result of the need for alternatives to native (prohibited) resources in spite of their great importance to this community. This suggests that a contextual analysis of this phenomenon must take into account not only the proportion of species used (native as well as introduced), but also local preferences.

The second question relates to the transmission of local knowledge. The enforced restriction of the access to native plants may result, in the long run, to the abandonment of their use, and thus a loss of local knowledge. This is particularly serious in the present study, as the most knowledgeable people are very old and the young have not shown much interest in this accumulated knowledge. This will surely affect the resilience of the local knowledge [25]. Additionally, the opportunities for these specialists to invent and experiment with new native resources will be lost, as well as the possibility for scientists to investigate new bio-active products. In agreement with observations in other Atlantic Forest communities [28-30], we consider that "the process of household Forest uses, of economic alternatives, and of environmental education may be closely tied and may serve as a vehicle for conservation, and key individuals from each community should be included in management programs" [25].

## Conclusion

This study demonstrated that local specialists in the Atlantic Forest community studied tend to agree with each other in terms of the plants used to treat blood-related problems, but cite a much more diverse group of plants to treat problems related to the respiratory and digestive systems – two important categories in studies undertaken in different parts of the world. In the case of the community examined here, the extensive repertory of plants employed to treat respiratory problems seems to be a response to problems related to the extensive use of fire in local agricultural practices. The local medicinal flora is largely based on plants that are either cultivated or obtained from anthropogenic zones, possibly due to the use and access restrictions of the legally protected neighboring forest. Despite these restrictions, the species with the highest use-value by this community was *Pithecellobium cochliocarpum *(Gomez) Macb., a native plant of the Atlantic Forest.

## Supplementary Material

Additional File 1 List of Plants used in the community of "Três Ladeiras" in the municipality of Igarassu (Pernambuco, Northeast Brazil): indications and Use Value. C = cultivated. NC = non-cultivated. Click here for file
