# Supplementary material for: Knowledge and use of medicinal plants by local specialists in an region of Atlantic Forest in the state of Pernambuco (Northeastern Brazil)
Source: J Ethnobiol Ethnomed. 2005 Nov 1;1:9. doi: 10.1186/1746-4269-1-9 (PMC1291389; doi:10.1186/1746-4269-1-9)
Supplement: Additional File 1 — List of Plants used in the community of "Três Ladeiras" in the municipality of Igarassu (Pernambuco, Northeast Brazil): indications and Use Value. C = cultivated. NC = non-cultivated. [file 1746-4269-1-9-S1.pdf]

Plants used in the community of “Três Ladeiras” in the municipality of Igarassu (Pernambuco, Northeast Brazil): indications and Use Value. C= cultivated. NC= non-cultivated.

Botanical family and taxon	name(s) recorded	Claimed medical use	Part(s) used	Use Value	Status
**Acanthaceae**					
Justicia pectoralis Jacq.	Chambá	Cough	Whole Plant	0.33	C
Graptophyllum pictum Griff.	Meracilina	Rash, cicatrizing	Leaves	0.67	C
**Amaranthaceae**					
Pfaffia glomerata (Spreng.) Pederson	Acônito	Fever	Leaves	0.67	C
**Anacardiaceae**					
Anacardium occidentale L.	Caju Roxo	Anti-inflammation, inflammation of the uterus	Bark of the stem	0.33	C
Mangifera indica L.	Manga Espada	Cough, anti-inflammation, fever	Leaves	0.83	C
Schinus terebinthifolius Raddi.	Aroeira	Anti-inflammation, cicatrizing	Bark of the stem and Leaves	1.17	C
**Annonaceae**					
Anonna marcgravii Mart.	Aticum	Sinusitis, tiredness, fever, expectoration	Leaves	0.83	NC
**Apiaceae**					
Pimpinella anisum L.	Erva doce	Dysentery, digestive	Seed and Leaves	0.33	C
**Apocynaceae**					
Hancornia speciosa Gomez	Mangaba	Pain in the testicle	Latex	0.17	C
**Arecaceae**					
Acrocomia intumescens Drude	Macaíba	Cough	Leaves	0.17	NC
Cocos nucifera L.	Coco Verde	Dysentery	Pericarp of the fruit	0.17	C

Cocos nucifera L.	Coco Vermelho	Hepatitis, problem of the kidney,	Liquid endosperm, Bark of the stem	0.50	C
Elaeis guineensis L.	Dendê	Boil	Oil	0.17	C
Syagrus sp.	Coco Catolé	Liver diseases, problem of the kidney	Root	0.50	NC
**Asteraceae**					
Acanthospermum hispidum DC.	Espinho de Cigano	Cough, dysentery	Root	0.67	NC
Coniza bonariensis (L.) Cronq.	Rabo de Raposa	Fungi	Leaves	0.33	NC
Egletes viscosa (L.) Less.	Macela	Swelling	Leaves	0.17	NC
Helianthus annuus L.	Girassol	Spill	Seed	0.17	C
Tagetes sp.	Cravo de Defunto	Cough	Flower	0.17	C
Vernonia condensata Baker	Alcachofra	Dysentery, liver diseases	Leaves	0.33	C
**Begoniaceae**					
Begonia vitifolia Schott.	Capeba	Digestive	Leaves	0.17	C
**Bignoniaceae**					
Tabebuia avellanedae Lor. et Gris	Pau D´arco Roxo	Rheumatic, cancer, column, spleen	Bark of the stem	0.67	NC
**Boraginaceae**					
Heliotropium indicum L.	Fedegoso	Derrame, digestive, spleen	Leaves e Root	0.50	NC
**Brassicaceae**					
Nasturtium officinale R. Br.	Agrião	Cough, problem of the kidney, flu	Whole Plant	0.83	NC
**Bromeliaceae**					
Tilandsia usneoides L.	Salambaia	Gastritis	Whole Plant	0.17	C
**Burseraceae**					
Protium heptaphyllum (Aubl.) March	Amescla	Headache,pain of tooth, flu,rheumatic	Resin	1.00	NC

**Caricaceae**					
Carica papaya L.	Mamão	Cough	Fruit	0.33	C
**Caesalpiniaceae**					
Caesalpinia ferrea Mart. Ex Tul.	Jucá	Rheumatic, cicatrizing Bruise	Fruit and Bark of the stem	0.50	NC
Hymenaea martiana Hayne	Jatobá	Bruise, anti-inflammation , rheumatic	Bark of the stem and Seed	1.33	NC
Senna occidentalis (L.) (Link)	Manjerioba	Cough	Flower and Root	0.17	NC
Tamarindus indica L.	Tamarindo	Problem of the kidney	Bark of the stem	0.17	C
Not identified	Pau D`óleo	Boil	Oil	0.17	?
Senna obtusifolia (L.) H.S. Irwin & Barnbey	Mata Pasto	Cough	Root and Leaves	0.17	NC
**Cecropiaceae**					
Cecropia palmata Willd	Imbaúba Branca	Anti-inflammation, inflammation of the uterus	Root	0.33	NC
**Capparaceae**					
Cleome spinosa Jacq.	Muçambê	Cough	Flower	0.17	NC
**Caprifoliaceae**					
Sambucus australis Cham. & Schlecht	Flor de Sabugo	Cough, measles	Flower	0.50	C
**Chenopodiaceae**					
Chenopodium ambrosioides L.	Mastruz	Cough, illness, worm,	Leaves	0.67	C
**Clusiaceae**					
Vismia guianensis (Aubl.) Choisy.	Lacre	Itch	Leaves	0.33	NC
Symphonia sp.	Bulandi	Dysentery	Leaves	0.17	NC

**Convolvulaceae**					
Ipomoea asarifolia (Ders.) R. et Sch	Salsa	Itch	Flower and Root	0.17	NC
Operculina alata (Ham.) Urb.	Batata de Pulga	Fever, flu	Root	0.50	NC
Not identified	Acanfor	Cough	Root	0.33	?
**Costaceae**					
Costus sp.	Cana de Macaco	Diabetes	Root	0.17	C
**Curcubitaceae**					
Luffa operculata Cong.	Cabacinho	Rheumatic	Fruit and Seed	0.17	NC
Sechium edule (Jacq.) Sw.	Chuchu	Cough	Fruit	0.17	C
**Euphorbiaceae**					
Croton sp.	Marmeleiro	Renal problems	Bark of the stem	0.17	NC
Cnidoscolus urens (L.) Arthur	Urtiga Branca	Anti-inflammation, cough	Root	0.50	NC
Euphorbia thymifolia L.	Rins/Pé de Pombo	Problem of the kidney, Dysentery	Whole Plant	0.67	NC
Phyllanthus niruri L.	Quebra-pedra	Problem of the kidney	Root	0.17	NC
**Fabaceae**					
Bowdichia virgilioides Kunth	Sucupira Branca	Anti-inflammation, inflammation of the uterus	Bark of the stem and Root	0.50	NC
Zornia diphyla (L.) Pers.	Urinana		Whole plant	0.17	NC
**Iridaceae**					
Crocus sp.	Açafrão	Bruise	Fruit	0.33	C
**Lamiaceae**					
Aeolanthus suaveolens Mart. ex Spreng.	Macaçá	Pain, pain of ear, headache	Leaves	1.33	C
Mentha pulegium L.	Vick	Cough	Leaves	0.17	C

Mentha sp.	Hortelã Miúdo	Colic, worm, faint, cough,	Leaves	0.83	C
Ocimum basilicum L.	Manjericão	Sight	Leaves	0.50	C
Ocimum gratissimum L.	Alfavaca deCaboclo	Sinusitis	Leaves	0.17	C
Ocimum gratissimum L.	Louro/Alfavaca	Cough	Leaves	0.17	C
Ocimum minimum L.	Manjericão Miúdo	Bruise	Leaves	0.17	C
Plectranthus barbatus Andr.	Boldo	Dysentery	Leaves	0.17	C
Plectranthus sp.	Hortelã do Maranhão	Headache, colic, pain in the articulation	Leaves	0.50	C
Plectranthus sp	Hortelã Bahia	Cough, pain of ear	Leaves	0.83	C
Rosmarinus officinalis L.	Alecrim	Pain	Leaves	0.17	C
Not identified	Hortelã Pastilha	Cough, pain	Whole Plant and Leaves	0.50	?
Not identified	Verga Morta	Pain	Whole Plant and Leaves	0.50	?
**Lauraceae**					
Persea americana Mill.	Abacate	Dysentery, problem of the kidney, liver diseases	Leaves	0.83	C
**Lecythidaceae**					
Eschweilera ovata (Cambess.) Miers	Embiriba	Cicatrizing	Bark of the stem	0.33	NC
**Mimosaceae**					
Pithecellobium cochliocarpum (Gomez) Macbr	Barbatenon	Cicatrizing, illness, inflammation of the uterus	Bark of the stem and Latex	1.83	NC
**Liliaceae**					
Allium aescalonicum L.	Cebola Branca	Cough, pain, digestive	Bulb	0.67	C
Allium sativum L.	Alho	Derrame, cough	Bulb	0.33	C
Aloe vera (L.) Burm.	Babosa	Gastritis, boil, anti-inflammation	Leaves	0.83	C
Eleutherine plicata Herb.	Alho do Mato	Cough, flu	Bulb	0.67	C

**Loranthaceae**					
Phthirusa pyrifolia (H.B.K.) Eichl.	Estanca Sangue	Anti-inflammation, pain of tooth,	Leaves	0.33	NC
Phthirusa pyrifolia (H.B.K.) Eichl.	Esterco de Passarinho	Pain of tooth	Whole Plant	0.50	NC
**Malvaceae**					
Gossypium sp.	Algodão Crioulo	Headache, pain of neck swelling	Leaves	0.50	C
Malva sp.	Malva Branca	Swelling	Whole Plant	0.17	NC
Urena lobata L.	Malva Rosa	Fever, expectoration,	Leaves	0.33	NC
**Melastomataceae**					
Not identified	Tonga-tonga	Pain of tooth,	Seed and Leaves	0.17	?
**Moraceae**					
Dorstenia sp.	Batata Contra Erva	Cough, expectoration, bronquitis	Root	0.83	NC
**Musaceae**					
Musa paradisiaca L.	Banana-prata	Cough	Flower	0.33	C
**Myristicaceae**					
Not identified	Urucuba	Pain, dysentery	Seed	0.50	?
**Myrtaceae**					
Eucalyptus citriodora Hook	Eucalipto	Fever	Leaves	0.17	C
Eugenia uniflora L.	Pitanga	Dysentery	Leaves	0.17	C
Psidium cattleyanun Sabine	Araçá	Dysentery	Leaves	0.17	C
Psidium guajava Raddi	Goiaba	Dysentery	Leaves	0.17	C
Syzygium jambolanum (Lam.) DC	Azeitona Roxa	Diabetes	Bark of the stem	0.17	C
Not identified	Joboticaba	Sight, digestive	Bark of the stem and Fruit	0.33	?

**Nyctaginaceae**					
Boerhaavia diffusa L.	Pega-pinto	Anti-inflammation, Cough	Root	1.17	NC
**Olacaceae**					
Ximenia americana L.	Ameixa	Rash	Bark of the stem and Leaves	0.17	NC
**Oxalidaceae**					
Averrhoa carambola L.	Carambola	Problem of the kidney	Leaves and Fruit	0.83	C
**Papaveraceae**					
Argemone mexicana L.	Cardo Santo	Spill	Whole Plant and Seed	0.50	N
**Passifloraceae**					
Passiflora edulis Sims.	Maracujá	Diabetes	Bark of the stem	0.17	C
**Pedaliaceae**					
Sesamum orientale L.	Gergelim Preto	Spill	Whole Plant	0.17	C
**Phytolaccaceae**					
Petiveria alliacea L.	Atipim	Swelling, rheumatic, pain	Leaves and Root	0.67	C
**Poaceae**					
Brachiaria mutica (Forsk.) Stapf	Capim de Planta	Anti-inflammation, inflammation of the uterus	Root	0.33	C
Cymbopogon citratus (DC.) Stapf	Capim Santo	Dysentery, tranquillizer	Leaves and Root	0.50	C
Dendrocalamus giganteus Munro	Bambu	Column	Root	0.17	C
Phalaris canariensis L.	Alpiste	Hepatitis	Seed	0.17	C
Saccharum officinarum L.	Cana	Diabetes	Leaves	0.17	C
**Rhamnaceae**					
Ziziphus joazeiro Mart.	Juá	Cough, illness, louse	Bark of the stem	0.83	NC

**Rhizophoraceae**					
Rhizophora mangle L.	Mangue	Anti-inflammation, inflammation of the uterus	Flower	0.33	NC
**Rosaceae**					
Rosa sp	Rosa	Heart, cough	Flower	0.33	C
**Rubiaceae**					
Borreria verticillata (L.) G. Mey	Vassoura de Botão	Dysentery, anti-inflammation	Whole Plant	1.17	NC
Cephaelis ipecacuanha (Brot.) A. Rich.	Papeconha	Rash, sight, cough	Root	0.50	NC
**Rutaceae**					
Citrus sinensis (L.) Osbeck	Laranja	Insomnia, dysentery	Flower and Leaves	0.83	C
Pilocarpus sp.	Jaborandi	Dysentery, pain of tooth,	Leaves and Root	0.33	NC
Ruta graveolens L.	Arruda	Pain of ear , pain in the body, colic	Leaves	0.83	C
**Sapindaceae**					
Cardiospermum halicacabum L.	Cipó de Vaqueiro	Bruise	Whole Plant	0.33	NC
Cupania sp.	Acabatã do Rêgo	Anti-inflammation, inflammation of the uterus	Bark of the stem	0.33	NC
**Sapotaceae**					
Pradosia sp.	Burinhê	Tiredness, expectoration	Bark of the stem and oil	0.33	NC
**Scrophulariaceae**					
Scoparia dulcis L.	Vassourinha	Bruise	Whole Plant	0.17	NC
**Smilacaceae**					
Smilax sp.	Japecanga	Anti-inflammation	Whole Plant	0.17	NC

**Solanaceae**					
Solanum americanum Mill.	Erva Moura	Itch	Leaves and Root	0.17	NC
Solanum panicultum L.	Jurubeba Branca	Hepatitis, anaemia,<br>spleen, digestive, problem of the kidney	Seed, Root and Fruit	1.00	NC
**Turneraceae**					
Turnera ulmifolia L.	Chanana	Cough	Flower	0.17	NC
**Verbenaceae**					
Lantana camara L.	Chumbinho	Cough	Flower	0.33	NC
Lippia alba (Mill.) N.E. Br.	Erva Cidreira	Anaemia, digestive	Leaves	0.67	NC
Stachytarpheta elatior Schrad.	Mocotó	Cough, flu	Root	0.67	NC
Vitex agnus-castus L.	Liamba	Fever	Leaves	0.17	C
**Vitaceae**					
Cissus sp.	Insulina	Diabetes	Leaves	0.17	C
**Zingiberaceae**					
Alpinia zerumbet (Pers.) Burt. R. M. Smith	Colônia	Flu, fever, headache, cough	Leaves, flower and root	1.67	C
Not identified 01	Caldeiro	Cough, problem of the kidney	Leaves	0.33	?
Not identified 02	Cravo branco	Heart, blood pressure	Flower	0.33	?
Not identified 03	Gramineira	Pain of tooth,	Latex	0.17	?
Not identified 04	Imbira Branca	Pain	Seed	0.17	?
Not identified 05	Jaracatiá	Worm, gastritis	Latex and Fruit	0.33	?
Not identified 06	Má de Cravo	Pain, colic	Leaves	0.17	?
